# A cross-sectional study reporting concussion exposure, assessment and management in Western Australian general practice

**DOI:** 10.1186/s12875-021-01384-1

**Published:** 2021-03-02

**Authors:** Elizabeth Thomas, HuiJun Chih, Belinda Gabbe, Melinda Fitzgerald, Gill Cowen

**Affiliations:** 1 School of Public Health, Curtin University, Bentley, Australia; 2 Division of Surgery, Faculty of Health and Medical Sciences, The University of Western Australia, Perth, Australia; 3 Centre for Clinical Research Excellence, Curtin University, Bentley, Australia; 4 School of Public Health and Preventive Medicine, Monash University, Melbourne, Australia; 5 Health Data Research UK, Swansea University Medical School, Swansea University, Swansea, UK; 6 Curtin Health Innovation Research Institute, Curtin University, Bentley, Australia; 7 Perron Institute for Neurological and Translational Science, Sarich Neuroscience Research Institute Building, Nedlands, Australia; 8 Curtin Medical School, Curtin University, Bentley, Australia

## Abstract

**Background:**

General Practitioners (GPs) may be called upon to assess patients who have sustained a concussion despite limited information being available at this assessment. Information relating to how concussion is actually being assessed and managed in General Practice is scarce. This study aimed to identify characteristics of current Western Australian (WA) GP exposure to patients with concussion, factors associated with GPs’ knowledge of concussion, confidence of GPs in diagnosing and managing patients with concussion, typical referral practices and familiarity of GPs with guidelines.

**Methods:**

In this cross-sectional study, GPs in WA were recruited via the RACGP WA newsletter and shareGP and the consented GPs completed an electronic survey. Associations were performed using Chi-squared tests or Fisher’s Exact test.

**Results:**

Sixty-six GPs in WA responded to the survey (response rate = 1.7%). Demographics, usual practice, knowledge, confidence, identification of prolonged recovery as well as guideline and resource awareness of GPs who practised in regional and metropolitan areas were comparable (*p *> 0.05). Characteristics of GPs were similar between those who identified all symptoms of concussion and distractors correctly and those who did not (*p *> 0.05). However, 84% of the respondents who had never heard of concussion guidelines were less likely to answer all symptoms and distractors correctly (*p *= 0.039). Whilst 78% of the GPs who were confident in their diagnoses had heard of guidelines (*p *= 0.029), confidence in managing concussion was not significantly associated with GPs exposure to guidelines. It should be noted that none of the respondents correctly identified signs of concussion and excluded the distractors.

**Conclusions:**

Knowledge surrounding concussion guidelines, diagnosis and management varied across GPs in WA. Promotion of available concussion guidelines may assist GPs who lack confidence in making a diagnosis. The lack of association between GPs exposure to guidelines and confidence managing concussion highlights that concussion management may be an area where GPs could benefit from additional education and support.

**Supplementary Information:**

The online version contains supplementary material available at 10.1186/s12875-021-01384-1.

## Background

Sports-related concussion (SRC) has been the focus of increased publicity in recent years as a result of attention in the lay press, however, SRC accounts for approximately only 20% of concussion in the general population [1, 2]. Other major causes of concussion include falls, motor vehicle and bicycle crashes, and assault, [3–6] with falls the leading cause in older populations [7]. General Practitioners (GPs) are often called upon to assess patients who have sustained a concussion and historical notes or a recent discharge summary from the Emergency Department may be unavailable at this assessment. The GP may, or may not, know the patient, and it may, or may not, be clear to the GP whether the patient’s presenting symptoms originate from the described concussive injury. This complicates a GP’s understanding of how a patient is affected by a concussion and limits their ability to optimise early clinical assessment and management with a view to improving long term outcomes.

The diagnosis of concussion is clinical and is made following assessment by a medical practitioner. There are currently no blood tests or imaging techniques available to GPs to confirm a diagnosis of concussion [8]. Symptoms such as dizziness, fogginess, headache, nausea, sleep and mood disturbance, memory impairment and neck pain are indicators of concussion [9] after either a direct blow to the head, or after sustaining a transmitted force to the head from an impact to the body [10]. Collateral history may also assist with diagnosis, with brief loss of consciousness, unsteadiness, posturing, a dazed appearance, disorientation and agitation all suggestive of concussion [11].

Once a GP has excluded focal neurology, a diagnosis of concussion is considered. After making such a diagnosis a GP must then address how the patient can be best managed and make decisions around the need for involvement of other health care professionals in the patient’s management. Typically, concussed adults recover in 10–14 days [12, 13]. Normal recovery from concussion in children can take up to 28 days [14–17]. Approximately 85% of patients will recover within these timeframes [18, 19].

The role of the GP is pivotal to the patient’s safe return to activities of daily living (ADLs), and includes decisions around screen time, reading, exercise, school and learning, work, driving, sport, and the potential need for other intervention. Research providing support for these decisions can be difficult to interpret, contradictory, complex and difficult to implement, [20, 21] especially outside return to sport protocols [22]. Furthermore, there is a potential risk of slowing recovery time, or exacerbating patient condition, by failing to optimise management [23] or by returning to ADLs too soon [24]. This creates a clinical environment prone to variation in care between clinicians and between institutions or jurisdictions, particularly outside SRC management. Information relating to how concussion is being managed is scarce, and it is unknown whether the care is optimal and whether the impost on the health care system can be reduced simply by improved medical education.

There is a paucity of Australian literature exploring GPs’ exposure to patients presenting with concussion. This study aimed to identify characteristics of current Western Australian (WA) GP exposure to patients with concussion, factors associated with their knowledge, confidence in diagnosing and managing patients with concussion, referral practices and familiarity with guidelines. Such information is necessary to identify the challenges faced by GPs relating to concussion diagnosis and management. Identification of such challenges is crucial for targeting areas where GPs require increased support and improved GP education, with the ultimate goal of improving patient care.

## Methods

### Study design and sample recruitment

This cross-sectional survey of WA GPs received ethics approval from Curtin University Ethics Committee (Approval Number HREC2019-0602). GPs were recruited using a convenience sample over a two-month period. To be included in this study, GPs were required to be working in a clinical role in a general practice setting within WA.

### Data collection

An electronic survey was developed to determine the current approach to concussion diagnosis and management in general practice in WA (Supplementary Text 1). The study investigators assessed the readability and content of the questionnaire to assure its face and content validity. Key areas were identified from current literature [25, 26]. Demographics of the respondents, as well as their reported exposure to, and identification of, concussion diagnoses were included, as were questions relating to GP knowledge of symptoms and signs commonly identified as potential flags for a concussion diagnosis encompassing all “sub-types” of concussion [27]. The survey was disseminated via the Royal Australian College of General Practitioners (RACGP) WA newsletter and the WA ShareGP group.

The survey (Supplementary Text 1) was intended to briefly assess:

1. scope of practice. This included age and professional level of the doctor, additional post graduate qualifications and special interest affiliations, hours worked, practice location (metropolitan/regional) and work outside general practice.
2. exposure to patients with potential/confirmed concussion and frequency of diagnosis.
3. knowledge of potential symptoms of concussion: possible symptoms and distractors were offered (Supplementary Text 2a).
4. knowledge of potential clinical signs of concussion: possible signs and distractors were offered (Supplementary Text 2b).
5. confidence in diagnosis, management, and identification of prolonged recovery
6. follow up and referral patterns
7. familiarity with any concussion guideline(s) or assessment tool(s)
8. use of clinical coding in General Practice

### Data analyses

Demographic characteristics, usual practice and knowledge of the GPs were compared by practice location: mean and standard deviation were reported for continuous variables whilst frequency and percentage were reported for categorical variables. Independent t-tests were performed to compare group means for normally distributed continuous variables. Associations between categorical variables and practice location, as well as knowledge, confidence and practices were performed using Chi-square tests, or Fisher’s Exact test when there was low number of expected frequencies. The significance level was set at 0.05. Data were analysed using Stata 14/IC (Stata Corp, Texas).

## Results

### Characteristics, usual practice and knowledge of the GP respondents

The survey was disseminated to 3880 WA GPs. Sixty-six GPs responded. The response rate was 1.7%. The respondent furthest north was from Karratha and furthest south from Albany. Of the respondents one was a GP registrar and 12 held dual Fellowships of the Australian College of Rural and Remote Medicine (ACRRM) and the RACGP. Eight respondents (12%) had additional post-graduate level Sports Medicine qualifications (Certificate/Diploma in Sports Medicine). Twenty-one respondents (32%) reported also working outside their general practice surgery setting. None of the respondents were members of the Sports and Exercise Medicine or Musculoskeletal Medicine RACGP Specific Interest groups.

The demographics of GPs who practised in regional and metropolitan areas were comparable (*p *> 0.05) (Table 1). Diagnoses made, referral practices, knowledge of symptoms and signs of concussion, confidence in diagnosis and management, identification of prolonged recovery, and guideline and resource awareness were also comparable between GPs who practised in regional and metropolitan areas (Table 1).

**Table 1:** Demographic, usual practice and knowledge of the GP, by location of practice (*n *= 64^a^)

	Regional area (*n *= 15)	Metropolitan area (*n *= 49)	*p-*value^#^
Hours worked at GP, *mean *± *standard deviation*	28.9 ± 12.9	32.7 ± 9.1	0.21
Age groups (years old), *n (%)*			0.48
- ≤ 45	7 (46.7)	28 (57.1)	
- > 45	8 (53.3)	21 (42.9)	
Work outside sessional load, *n (%)*			0.99^^^
- No	10 (66.7)	34 (69.4)	
- Yes	5 (33.3)	15 (30.6)	
Concussion diagnoses made/year, *n (%)*			0.15
- < 5	7 (46.7)	33 (67.4)	
- ≥ 5	8 (53.3)	16 (32.7)	
Frequency of concussion as a secondary diagnosis, *n (%)*			0.32^^^
- Never/Rarely	10 (66.7)	39 (79.6)	
- About half the time/Frequently	5 (33.3)	8 (20.4)	
Would refer to specialist, *n (%)*			0.57
- No	8 (53.3)	22 (44.9)	
- Yes	7 (46.7)	27 (55.1)	
Would refer for diagnostic imaging, *n (%)*			0.10
- No	11 (73.3)	23 (48.9)	
- Yes	4 (26.7)	24 (51.1)	
Identified all symptoms and distractors correctly, *n (%)*			0.25
- No	8 (53.3)	34 (69.4)	
- Yes	7 (46.7)	15 (30.6)	
Identified all signs and distractors correctly, *n (%)*			-
- No	15 (100.0)	49 (100.0)	
- Yes	0	0	
Confident in making a diagnosis of concussion, *n (%)*			0.99^^^
- No	3 (20.0)	9 (18.4)	
- Yes	12 (80.0)	40 (81.6)	
Confident in managing a diagnosis of concussion, *n (%)*			0.70
- No	5 (33.3)	19 (38.8)	
- Yes	10 (66.7)	30 (61.2)	
Understanding of prolonged recovery in concussed adults			0.99^^^
- > 5 days	9 (60.0)	28 (57.1)	
- > 14 days	6 (40.0)	19 (38.8)	
- > 28 days	0	2 (4.1)	
Understanding of prolonged recovery in concussed children/adolescents			0.55^^^
- > 5 days	11 (73.3)	41 (83.7)	
- > 14 days	2 (13.3)	5 (10.2)	
- > 28 days	2 (13.3)	3 (6.1)	
Ever heard of guidelines, *n (%)*			0.35^^^
- No	6 (40.0)	13 (26.5)	
- Yes	9 (60.0)	36 (73.5)	
Have a protocol for coding, *n (%)*			0.99
- No	10 (66.7)	32 (66.7)	
- Yes	5 (33.3)	16 (33.3)	

^a^Two participants did not report their practice location

^#^ Difference in mean hours worked between GP who worked in regional or metropolitan area was assessed using independent-samples t-test. Associations between categorical demographic variables and regional or metropolitan area were assessed using Chi-squared tests (or Fisher’s exact tests^^^ due to low number of expected frequencies)

Approximately 45% of the respondents reported requesting imaging to diagnose a concussion. Despite 81% of respondents reporting feeling confident making a concussion diagnosis, only 63% felt confident to manage this diagnosis (Table 1).

A lack of clarity around what constitutes a delayed recovery was also identified (Table 1). When patients were deemed to be “slow to recover”, close observation and referral on to a specialist were identified as management strategies for 53% of responders. Thirty-five responders identified their referral pathways; the most commonly identified was the emergency department (38%), followed by sports medicine clinic (25%), physiotherapist (18%), neurologist (13%), neurosurgeon (5%) and concussion clinic (3%).

### Association between annual number of concussion diagnoses and frequency of concussion as a secondary diagnosis

Among the GPs who made less than five concussion diagnoses per year, most (78%) *never o*r *rarely* made a diagnosis of concussion as a secondary diagnosis (Table 2). Similarly, most GPs who made more than five concussion diagnoses per year (77%) *never o*r *rarely* diagnosed concussion as a secondary diagnosis. There was no association between the number of concussion diagnoses made per year and the frequency of concussion as a secondary diagnosis (Table 2).

**Table 2:** GP concussion diagnoses per year and frequency of concussion as a secondary diagnosis (*n* = 66)

Concussion diagnoses made/year	Frequency of concussion as a secondary diagnosis		*p-*value^
	Never/ Rarely	About half the time/ Frequently	0.96
- < 5	31 (60.8%)	9 (60.0%)	
- ≥ 5	20 (39.2%)	6 (40.0%)	

^^^Association between number of concussion diagnoses a GP made per year and the frequency of concussion as a secondary diagnosis was assessed using Chi-squared test

### Symptom and Sign Identification

All respondents correctly identified headache, fogginess, dizziness, difficulty concentrating, irritability and drowsiness or sleep disturbance as potential symptoms of concussion. Sixty-three respondents (96%) identified sensitivity to light or sound as a possible symptom, 62 (94%) identified nausea and vomiting as potential symptoms and 41 (63%) identified neck pain as potentially important when making a concussion diagnosis. Twenty-three (35%) respondents both correctly identified all offered symptoms of concussion and correctly excluded the distractors (Table 3).

**Table 3:** Associations between GP characteristics and correct symptom and sign identification (*n* = 66)

GP characteristics	Identified all symptoms and distractors correctly		*p-*value^#^	Identified all signs and distractors correctly		*p-*value^*^
	No (*n *= 43)	Yes (*n *= 23)		No (*n *= 66)	Yes (*n *= 0)	
Hours worked at GP, *mean* ± *standard deviation*	30.7 ± 10.8	34.2 ± 8.3	0.18	31.9 ± 10.0	-	-
Age groups (years old), *n (%)*			0.64			-
- ≤ 45	25 (58.1)	12 (52.2)		37 (56.1)	0	
- > 45	18 (41.9)	11 (47.8)		29 (43.9)	0	
Location of GP practice, *n (%)*			0.25			-
- Regional	8 (19.1)	7 (31.8)		15 (23.4)	0	
- Metropolitan	34 (81.0)	15 (68.2)		49 (76.6)	0	
Concussion diagnoses made/year, *n (%)*			0.31			-
- < 5	28 (65.1)	12 (52.2)		40 (60.6)	0	
- ≥ 5	15 (34.9)	11 (47.8)		26 (39.4)	0	
Work outside sessional load, *n (%)*			0.78			-
- No	30 (69.8)	15 (65.2)		45 (68.2)	0	
- Yes	13 (30.2)	8 (34.8)		21 (31.8)	0	
Ever heard of guidelines, *n (%)*			0.039			-
- No	16 (37.2)	3 (13.0)		19 (28.8)	0	
- Yes	27 (62.8)	20 (87.0)		47 (71.2)	0	
Have a protocol for coding, *n (%)*			0.75			-
- No	29 (69.1)	15 (65.2)		44 (67.7)	0	
- Yes	13 (31.0)	8 (34.8)		21 (32.3)	0	

^#^ Difference in mean hours worked between GPs who answered all symptoms and distractors correctly was assessed using independent-samples t-test. Associations between categorical demographic variables and answering all symptoms and distractors correctly were assessed using Chi-squared tests

^*^Test of association could not be performed due to lack of GPs answering all the signs and distractors of concussion correctly

Characteristics of GPs were similar between those who identified all symptoms of concussion and distractors correctly and those who did not (Table 3). However, familiarity with guidelines was associated with concussion knowledge; eighty-four percent of GPs who were unaware of any guidelines were less likely to answer all symptoms and distractors correctly whilst 87% of the GPs who correctly identified all symptoms of concussion and distractors were aware of guidelines (Table 3).

Sixty-four respondents correctly identified balance disturbance, vestibular-ocular impairment and objective memory impairment as signs of concussion. Sixty-three respondents (98%) suspected concussion when presented with a facial or scalp injury. Forty-six respondents (72%) identified exercise intolerance and 45 (70%) identified orthostatic hypotension as potential signs of concussion. Forty-three respondents (67%) would be suspicious of concussion in a patient with neck tenderness. Twenty-three respondents (35%) identified all positive signs of concussion but no respondents also correctly identified all distractors (Table 3). As such, test of association could not be performed.

### Associations between GP characteristics and diagnostic confidence as well as referral practices

GPs’ hours worked, age group, professional level, location of practice, having work outside sessional load and having a protocol of coding were comparable between those who were, and were not, confident in diagnosing concussion (Table 4). However, the number of concussion diagnoses a GP made per year was associated with their confidence in diagnosing concussion (Table 4). Ninety-two percent of the GPs who were not confident in diagnosing concussion made less than five concussion diagnoses per year (Table 4). Having heard of concussion guidelines was also significantly associated with confidence in diagnosing concussion, with 78% of the GPs who were confident in their diagnoses aware of guidelines whilst 58% of the GPs who were not confident in their diagnoses having never heard of concussion guidelines (Table 4).

**Table 4:** Associations between GP characteristics and their confidence in diagnosing and managing concussion (*n *= 66)

GP characteristics	Confident in diagnosing		*p-*value^#^	Confident in managing		*p-*value^#^
	No (*n *= 12)	Yes (*n *= 54)		No (*n *= 24)	Yes (*n *= 42)	
Hours worked at GP, *mean *± *standard deviation*	34.1 ± 8.8	31.5 ± 10.3	0.42	29.7 ± 7.6	33.2 ± 11.1	0.17
Age groups (years old), *n (%)*			0.86			
- ≤ 45	7 (58.3)	30 (55.6)		18 (75.0)	19 (45.2)	0.019
- > 45	5 (41.7)	24 (44.4)		6 (25.0)	23 (54.8)	
Location of GP practice, *n (%)*			0.99^^^			0.70
- Regional	3 (25.0)	12 (23.1)		5 (20.8)	10 (25.0)	
- Metropolitan	9 (75.0)	40 (76.9)		19 (79.2)	30 (75.0)	
Concussion diagnoses made/year, *n (%)*			0.021^^^			0.020
- < 5	11 (91.7)	29 (53.7)		19 (79.2)	21 (50.0)	
- ≥ 5	1 (8.3)	25 (46.3)		5 (20.8)	21 (50.0)	
Work outside sessional load, *n (%)*			0.31^^^			0.15
- No	10 (83.3)	35 (64.8)		19 (79.2)	26 (61.9)	
- Yes	2 (16.7)	19 (35.2)		5 (20.8)	16 (38.1)	
Ever heard of guidelines, *n (%)*			0.029^^^			0.081
- No	7 (58.3)	12 (22.2)		10 (41.7)	9 (21.4)	
- Yes	5 (41.7)	42 (77.8)		14 (58.3)	33 (78.6)	
Have a protocol for coding, *n (%)*			0.74^^^			0.38
- No	7 (63.6)	37 (68.5)		14 (60.9)	30 (71.4)	
- Yes	4 (36.4)	17 (31.5)		9 (39.2)	12 (28.6)	

^#^ Difference in mean hours worked between GPs who were confident in diagnosing concussion was assessed using independent-samples t-test. Associations between categorical demographic variables and confidence in diagnosing concussion were assessed using Chi-squared tests (or Fisher’s exact tests^ due to low number of expected frequencies). The same statistical approach was applied for confidence in managing concussion

The number of concussion diagnoses a GP made per year was also associated with their confidence in managing concussion (Table 4). Seventy-nine percent of the GPs who were not confident in managing concussion made less than five concussion diagnoses per year. Age group of the GPs was associated with their confidence in managing concussion with 75% of the GPs who were not confident aged 45 years old or younger, whilst 55% of the GPs who were confident aged over 45 years old (Table 4). Contradictory to confidence in diagnosing concussion, confidence in managing concussion was not significantly associated with GPs exposure to guidelines (Table 4).

Characteristics of GPs were similar between the group who would refer for diagnostic imaging and the group who would not (Table 5). Similarly, characteristics of GPs were comparable between the group who would refer to specialist and the group who would not (Table 5).

**Table 5:** Associations between GP characteristics and referral for diagnostic imaging (*n *= 64) and to a specialist (*n *= 66)

GP characteristics	Referral for diagnostic imaging		*p-*value^#^	Referral to specialist		*p-*value^#^
	No (*n *= 35)	Yes (*n *= 29)		No (*n *= 31)	Yes (*n *= 35)	
Hours worked at GP, *mean* ± *standard deviation*	32.1 ± 11.0	32.1 ± 9.3	0.98	34.5 ± 10.5	29.7 ± 9.2	0.05
Age groups (years old), *n (%)*			0.32			0.10
- ≤ 45	17 (48.6)	18 (62.1)		14 (45.2)	23 (65.7)	
- > 45	18 (51.4)	11 (37.9)		17 (54.8)	12 (34.3)	
Location of GP practice, *n (%)*			0.10			0.57
- Regional	11 (32.4)	4 (14.3)		8 (26.7)	7 (20.6)	
- Metropolitan	23 (67.7)	24 (85.7)		22 (73.3)	27 (79.4)	
Concussion diagnoses made/year, *n (%)*			0.69			0.06
- < 5	20 (57.1)	18 (62.1)		15 (48.4)	25 (71.4)	
- ≥ 5	15 (42.9)	11 (37.9)		16 (51.6)	10 (28.6)	
Work outside sessional load, *n (%)*			0.78			0.55
- No	23 (65.7)	20 (69.0)		20 (64.5)	25 (71.4)	
- Yes	12 (34.3)	9 (31.0)		11 (35.5)	10 (28.6)	
Ever heard of guidelines, *n (%)*			0.38			0.30
- No	12 (34.3)	7 (24.1)		7 (22.6)	12 (34.3)	
- Yes	23 (65.7)	22 (75.9)		24 (77.4)	23 (65.7)	
Have a protocol for coding, *n (%)*			0.47			0.99
- No	22 (62.9)	20 (71.4)		21 (67.7)	23 (67.7)	
- Yes	13 (37.1)	8 (28.6)		10 (32.3)	11 (32.4)	

^#^ Difference in mean hours worked between GP who referred for diagnostic imaging was assessed using independent-samples t-test. Associations between categorical demographic variables and referral for diagnostic imaging were assessed using Chi-squared tests. The same statistical approach was applied for referral to specialist

### Associations between knowledge and confidence as well as referral practices and familiarity with guidelines

The proportion of GPs who did answer, or did not answer, all symptoms and distractors correctly was equally distributed between the group who were and were not confident in diagnosing as well as managing concussion (Table 6), and between referrers and non-referrers to a specialist (Table 7) or diagnostic imaging (Table 7). Answering all symptoms and distractors of concussion correctly was not associated with the GPs’ confidence in diagnosing or in managing concussion (Table 6). Answering all symptoms and distractors correctly was not associated with GPs’ referral to specialist, referral for diagnostic imaging (Table 7).

**Table 6:** Numbers of, and associations between knowledge and confidence (*n *= 66)

Concussion knowledge	Confident in diagnosing		*p-*value	Confident in managing		*p-*value
Identified all symptoms and distractors correctly, *n (%)*	No	Yes	0.52^^^	No	Yes	0.85^^^
- No	9 (75.0)	34 (63.0)		16 (66.7)	27 (64.3)	
- Yes	3 (25.0)	20 (37.0)		8 (33.3)	15 (35.7)	
Identified all signs and distractors correctly, *n (%)*			-			-
- No	12 (100.0)	54 (100.0)		24 (100.0)	42 (100.0)	
- Yes	0	0		0	0	

^^^Associations between knowledge and confidence in diagnosing concussion were assessed using Fisher’s exact test due to low number of expected frequencies. The same statistical approach was applied for confidence in managing concussion

**Table 7:** Associations between knowledge and referral practices (*n *= 66)

Concussion knowledge	Referral to specialist		*p-*value^#^	Referral for diagnostic imaging		*p-*value^#^
Identified all symptoms and distractors correctly, *n (%)*	No	Yes	0.26	No	Yes	0.83
- No	18 (58.1)	25 (71.4)		22 (62.9)	19 (65.5)	
- Yes	13 (41.9)	10 (28.6)		13 (37.1)	10 (34.5)	
Identified all signs and distractors correctly, *n (%)*			-			-
- No	31 (100.0)	35 (100.0)		35 (100.0)	29 (100.0)	
- Yes	0	0		0	0	

^#^Associations between knowledge and referral practices were assessed using Chi-squared tests

Eighty-two percent of the GPs who did not answer all signs and distractors of concussion correctly were confident in diagnosing concussion (Table 6). About 53% of the group who did, and 45% of the group who did not, answer all signs and distractors of concussion correctly would refer to a specialist, and diagnostic imaging, respectively (Table 7). However, due to lack of GPs answering all the signs of concussion correctly, the tests of association could not be performed.

## Discussion

In this study we examined the knowledge surrounding, and approach to, concussion diagnosis and management of GPs in WA. Overall, the findings suggest that knowledge and management practice amongst GPs is varied, albeit comparable between GPs who work in the metropolitan and regional areas. This supports previous literature where gaps in clinicians’ knowledge have been identified [28–33].

GPs are tasked with the provision of primary care to patients with both acute concussion and those with prolonged or persistent concussive symptoms. Failure to identify, diagnose or appropriately manage such presentations risks poorer outcomes. Likewise, the premature return of patients to ADLs where they may be at risk of further head injury poses a risk of second impact syndrome.

Only one GP reported being exposed to more than ten concussions per year whilst 61% of GPs reported exposure to less than five episodes per year. This may be because patients are failing to present for medical assessment following a potentially concussive injury or it may be due to failure of patients to present specifically to general practice. Alternatively, it is possible that a diagnosis of concussion may be overlooked when a patient does present and is assessed. Previous research has suggested that whilst unusual for more than 20 patients to be seen in a year by a family medicine specialist, approximately half see more than ten cases per year [34].

GPs play an important role in the acute phase of symptom management. Previous studies have highlighted the need for ongoing concussion education and awareness to best enable clinicians in this role and understanding the key features in assessment and management of concussion is essential for primary care providers [3]. 

In this study most respondents identified a delayed recovery as symptoms or signs persisting after five days in both adults and children. It has been demonstrated in experimental models that concussion injury triggers a neurometabolic cascade of events resulting in abnormal potassium, calcium, glutamate, glucose, and lactate levels and altered cerebral blood flow which takes seven to ten days to resolve [35]. Additional microglial and inflammatory responses can continue for considerably longer than this initial metabolic cascade [36]. If GPs are expecting resolution of symptoms in a concussed patient within 5 days, it may be that patients are being allowed to return to activities where they risk sustaining a further concussive force too early and this may have clinical consequences. It has been suggested that phase of recovery should be considered in regards to treatment approaches: Acute (0–4 weeks), Post-Acute (4–12 weeks) and Persistent (> 3 months). [37].

It was rare for a secondary diagnosis of concussion to be made by a respondent. It may be that this is due to the lack of exposure to multi-trauma patients in general practice, or may be because patients present later when concussion has been initially overlooked and other trauma-related primary diagnoses have been made elsewhere. Alternatively, GP exposure to concussion may be predominantly in patients who are improving post-event and requesting further management advice and input regarding return to usual ADLS, that is the concussion was diagnosed elsewhere previously.

Thirty-five percent of respondents identified all symptoms of concussion and distractors correctly. It was unexpected that only 63% of respondents identified neck pain as a potential symptom of a concussive injury and only 67% identified neck tenderness as a suggestive sign. Given that concussion can occur as a result of a transmitted force from the body, neck symptoms and signs should be identified as an integral part of any concussion assessment. Education of GPs to assess for concussion in patients presenting with neck pain or tenderness after a potentially concussive injury may increase concussion diagnosis.

Whilst GPs correctly identified signs of balance disturbance, objective memory impairment, vestibular-ocular impairment and facial/scalp injury as a sign that may indicate concussive injury, symptoms of autonomic dysfunction were less commonly identified as potential signs. Increasing evidence relating to dysautonomia has emerged in recent years [38] and this has led to development of early subthreshold exercise programs which may improve patient recovery [39]. Increasing GP knowledge in this area may reduce the numbers of patients suffering from prolonged symptoms and as such should be included in future GP concussion education programs.

Concussion has been described as having multiple symptoms and signs which can be divided into different ‘sub-types’ or clusters [40]. Diagnostic confidence and confidence in management was seen to increase with increasing exposure to patients with concussion in our study. The variation in concussion presentation means that no one strategy is appropriate for all [41] and may explain why older GPs were more confident in management of concussion. Further research is required to confirm that the confidence in management brought about with age is reflected in patient outcomes.

Concussion is a clinical diagnosis with no identifiable findings on standard CT or MRI protocols. Previous literature has suggested that, in clinical practice, cranial CT scanning is likely to be overused in the evaluation of mTBI [42]. It is suspected that doctors using imaging are doing so to exclude other brain injuries [43, 44] and subsequently making a diagnosis of concussion upon receipt of normal results. It may be that imaging is requested due to lack of confidence to clinically diagnose focal neurology, [45] or imaging may be driven by patient request, due to the threat of medico-legal repercussions of a missed alternative neurological diagnosis, or due to an alternative factor such as establishing a baseline in a person who may go on to sustain further concussions. It has been shown in youth concussion that utilisation of conventional neuroimaging results in identification of signs of TBI in 3.1% of cranial CT scans and 1.5% of MRI brain scans [46]. If the imaging in not being performed to exclude alternative diagnosis, this variation in GP care implies that there may be an unnecessary burden on the health economy relating to imaging that may not be justified. Further research clarifying why a GP chooses, or chooses not, to image a patient is required and what modality of imaging is chosen and why. The timing of imaging, if it is requested, also requires further investigation. Previous literature has identified that in paediatric concussion the majority of CT scans have been shown to be obtained during the acute concussion period, whereas MRI scans were ordered later in a patient’s recovery [47].

Failure to clarify these questions is a limitation of this study; prior to national distribution of this survey modification is required to allow this information to be gathered.

An awareness of concussion guidelines was associated with confidence in diagnosis but was not associated with confidence in management. Knowledge surrounding best practice relating to concussion management is rapidly evolving and best practice guidelines for SRC are drawn from the four yearly International Conference on Concussion in Sport’s Consensus Statement on Concussion in Sport. Seventy percent of respondents were familiar with at least one concussion guideline (Table 1), but only 28 respondents were familiar with the SCAT5 [48] and one was still using a SCAT3. [49] When concussions are sustained in a non-sporting environment inferences are drawn. There is a lack of clear guidance for general practitioners relating to management of concussion. Given that guideline awareness was linked to diagnostic confidence, our results suggest that there needs to be further dissemination of currently available guidelines [50, 51]. However, lack of link to confidence in management suggests that current guidelines may not be useful in this respect to GPs, and this is reflected in lack of information regarding when to allow patients, for example, to return to drive or work.

Ideally, nationally consistent, and regularly updated concussion diagnosis and management guidelines relevant to concussion from all causes, for all medical practitioners, including GPs, with links to appropriate resources may be one way of addressing the current inconsistencies in awareness. Education relating to management strategies appears to be an area of need. In a Medline search for “concussion” for 2019 alone there were 867 English language articles identified and it is possible that lack of confidence in this area is related to concussion research rapidly evolving in recent years.

Overseas literature has also highlighted gaps in concussion knowledge among family physicians, [52] and deficiencies relating to concussion guideline knowledge, as well as implementation of recommendations, in family physicians treating sports-related concussion [53]. Objective knowledge scores have been demonstrated not to predict self-reported concussion knowledge. [54].

Other than one missing data point, 21 GPs (33%) reported using clinical coding when recording a diagnosis of concussion in patient notes. This presents a problem when collecting and collating epidemiological data. GPs should be encouraged to code all consultations. This may require modification of current electronic medical record programs and ideally should allow for consistency of codes through different providers in both public and private health settings enabling further data regarding the incidence and prevalence to be collected. Previous literature has demonstrated that despite presentations to the emergency department being higher than those to outpatient departments with minor head injury, outpatient presentations were still significant [55]. Accurate GP coding of information is the first step, with subsequent routine data sharing which would facilitate further analysis for incidence estimates and research.

There is a risk of selection bias in any study which relies on participants to volunteer to respond to a questionnaire. Despite respondents coming from varying geographical locations across WA, our response rate was low and the findings need to be interpreted with caution. Whilst low response rate is not unusual in research involving GPs, strategies identified by Parkinson *et al* [56] (such as providing incentives and rewards, provision of paper format questionnaires with reply paid envelopes and contacting practice managers to encourage their general practitioners to complete the survey) will be incorporated into further national surveys with the aim of increasing response rate. Cross-sectional surveys may result in an over-representation of one group and this limitation is acknowledged, although none of the respondents were members of the sports and exercise or musculoskeletal medicine RACGP specific interest networks which may have been expected if this were the case. A further limitation is that the results reflected the knowledge and experience of each respondent on the day they completed the survey and the respondent may respond differently today.

It is perceived that the immediate priority for future attention is the development of further guidance for GPs in relation to diagnostic and management decisions in patients presenting with concussion from all causes, not just sport. Collection of data from GP Registrars will provide further information relating to current registrars’ knowledge in this area, and identification of concussion knowledge in newly qualified doctors will provide information to how concussion teaching and knowledge varies amongst medical schools. Data from rural and remote general practices, and Aboriginal Medical Services, are required in future studies to determine the differences between these practices and services, if any. In addition, clarification needs to be sought as to why imaging is requested by some GPs but not others and qualitative semi-structured interviews of GPs may be of value.

## Conclusion

Knowledge surrounding concussion guidelines, diagnosis and management varied across GPs in WA. Increasing the visibility of already available concussion guidelines to assist those GPs who are not confident making a diagnosis of concussion may result in increasing recognition of concussions presenting to general practice. The discrepancy between confidence in making a diagnosis of concussion and confidence in managing concussion indicates that GPs may need additional educational materials to assist in managing concussions. These materials may assist GPs working in metropolitan and regional areas with management of patients presenting early with concussion, and those with ongoing symptoms.

## Supplementary Information

**Additional file 1.**

**Additional file 2: Supplementary Text 2**. a. Symptoms offered to respondents to identify their knowledge of symptoms of concussion. b. Signs offered to respondents to identify their knowledge of signs of concussion.

## Data Availability

The datasets used and/or analysed during the current study are available from the corresponding author on reasonable request.

## Abbreviations

SRC: Sports-related concussion
GP: General Practitioner
GPs: General Practitioners
WA: Western Australia
ADLs: Activities of daily living
RACGP: Royal Australian College of General Practitioners
