# Supplementary material for: A cross-sectional study reporting concussion exposure, assessment and management in Western Australian general practice
Source: BMC Fam Pract. 2021 Mar 2;22:46. doi: 10.1186/s12875-021-01384-1 (PMC7927406; doi:10.1186/s12875-021-01384-1)
Supplement: Supplementary file 1 — Additional file 1. [file 12875_2021_1384_MOESM1_ESM.pdf]

Curtin University

Curtin University Medical School is conducting a research project to understand the approach to concussion diagnosis and management by General Practitioners. Turn over to complete a 5-minute survey, or follow the link below to do it online.

https://is.gd/concussion2019

Curtin University Ethics approval HREC2019-0602

About Concussion

Mild traumatic brain injury (mTBI), or concussion, accounts for 75-80% of all brain injuries in Australia and worldwide. While most concussion patients recover well within the first two weeks of injury, it is estimated that 10-20% will go on to experience persistent post-concussive symptoms (PCSS). There is a lack of standardisation in the approach to assessment and management of concussion in General Practice, and there is currently no way of knowing which patients are at risk of PCSS and require closer observation. The variation in care and lack of clarity as to the degree of care needed is likely to be cost-ineffective, and deprives patients of the best chance at making a timely complete recovery.

The Research

Our research is aimed at understanding current practice in managing concussions that present at General Practice clinics. The outcomes will be used to develop better guidelines and models of care to aid doctors in diagnosing and managing concussed patients.

You and your role

You have been invited to complete the survey because you are a General Practitioner and are likely to see and diagnose patients with concussive injuries. The survey includes questions about your professional qualifications, experience and the general area you practice, as well as your approach to diagnosing and managing concussion. Any data you provide cannot be linked back to your identity, and you can exit the survey at any point.

Disclaimer

This study is being undertaken by Curtin University researchers Dr Gill Cowen and Dr Libby Thomas. The study has received ethics approval from the Curtin Human Research Ethics Committee (approval number HRE2019-0602). If you would like to contact the research team directly, please do so have concerns with the study, please contact Curtin Ethics at hrec@curtin.edu.au

Consent

Your participation in this study is completely voluntary. You are not obliged to participate and may stop at any time. Your responses to this survey are strictly confidential and at no time will your answers be linked to your identity. To complete the survey will take approximately five minutes.

Are you willing to participate? ☐ Yes ☐ No

What is your age bracket (yrs)? ☐ 18-30 ☐ 30-45 ☐ >45

What is your professional level?

- ☐ GP
  - ☐ Post-graduate Certificate Sports Medicine
  - ☐ Diploma Sports Medicine
  - ☐ Master Sports Medicine
  - ☐ FACRRM
  - ☐ FRACGP
  - ☐ Other
- ☐ Registrar
- ☐ Other
  - ☐ RACGP SI MSK
  - ☐ RACGP SI SEM

How many hours do you routinely work in General Practice per week? ______________________

Do you do any work outside usual sessional load where concussions are encountered?

- ☐ Yes
  - ☐ Emergency Department
  - ☐ Sports cover
  - ☐ Aged Care
  - ☐ Other
- ☐ No

Where are you located in your General Practice (suburb or postcode)? ______________________

How many concussion diagnoses would you estimate you make per year?

- ☐ < 5
- ☐ 5-10
- ☐ 10-30
- ☐ 30-50

How often is concussion a secondary diagnosis?

- ☐ Never
- ☐ Rarely
- ☐ About half the time
- ☐ Frequently
- ☐ Almost always

In a concussion assessment would you refer for diagnostic imaging?

- ☐ Yes
  - ☐ MRI
  - ☐ CT
  - ☐ Other
- ☐ No

Would you consider any of these symptoms as indicative of a concussion?

- ☐ Headache
- ☐ Difficulty breathing
- ☐ Dizziness
- ☐ Fogginess
- ☐ Nausea/vomiting
- ☐ Sensitivity to light/sound
- ☐ Altered limb sensation
- ☐ Difficulty concentrating
- ☐ Chest pain
- ☐ Irritability
- ☐ Drowsiness or sleep disturbance
- ☐ Neck pain
- ☐ Other

Would any of these examination findings make you likely to suspect a patient has sustained a concussion?

- ☐ Orthostatic hypotension
- ☐ Hyporeflexia
- ☐ Facial or scalp injury
- ☐ Dysarthria
- ☐ Balance disturbance
- ☐ Vestibulo-ocular impairment
- ☐ Exercise intolerance
- ☐ Abnormal fundoscopy
- ☐ Abnormal cranial nerve examination
- ☐ Neck tenderness
- ☐ Objective memory impairment
- ☐ Altered visual acuity
- ☐ Other

Are you confident to make a diagnosis of concussion?

- ☐ Yes
- ☐ No

Are you confident to manage a concussed patient?

- ☐ Yes
- ☐ No

What would you consider a prolonged recovery from concussion in adults?

- ☐ >5 days
- ☐ >14 days
- ☐ >28 days
- ☐ >3 months

What would you consider a prolonged recovery from concussion in children/adolescents?

- ☐ >5 days
- ☐ >14 days
- ☐ >28 days
- ☐ >3 months

How do you manage those that don't settle quickly?

- ☐ Referral to a specialist — What kind? ______________________________
- ☐ Close observation
  - ☐ Weekly
  - ☐ Fortnightly
  - ☐ Monthly
- ☐ Other

Are you familiar with any current concussion guidelines, if so, which ones?

____

____

Are you familiar with any current concussion assessment tools?

____

____
