# Supplementary material for: A cross-sectional study reporting concussion exposure, assessment and management in Western Australian general practice
Source: BMC Fam Pract. 2021 Mar 2;22:46. doi: 10.1186/s12875-021-01384-1 (PMC7927406; doi:10.1186/s12875-021-01384-1)
Supplement: Supplementary file 2 — Additional file 2: Supplementary Text 2. a. Symptoms offered to respondents to identify their knowledge of symptoms of concussion. b. Signs offered to respondents to identify their knowledge of signs of concussion. [file 12875_2021_1384_MOESM2_ESM.docx]

**Supplementary Text 2**

1. *Symptoms offered to respondents to identify their knowledge of symptoms of concussion:*

| Symptoms offered to Respondents  (Grouped in Expected Response Categories) |  |
| --- | --- |
| **Suggestive of Concussion** | **Distractor** |
| Headache | Altered limb sensation |
| Dizziness | Difficulty breathing |
| Fogginess | Chest pain |
| Nausea and vomiting |  |
| Sensitivity to light/sound |  |
| Difficulty concentrating |  |
| Irritability |  |
| Drowsiness/sleep disturbance |  |
| Neck pain |  |

1. *Signs offered to respondents to identify their knowledge of signs of concussion:*

| Signs offered to Respondents  (Grouped in Expected Response Categories) |  |
| --- | --- |
| **Suggestive of Concussion** | **Distractor** |
| Orthostatic hypotension | Hyporeflexia |
| Facial/scalp injury | Dysarthria |
| Balance disturbance | Abnormal fundoscopy |
| Vestibular-ocular impairment | Altered visual acuity |
| Exercise intolerance |  |
| Neck tenderness |  |
| Objective memory impairment |  |
